# Short-Term Safety and Healthcare Utilization Following Intragastric Balloon Placement in Patients With and Without Diabetes Mellitus: A Propensity-Matched Analysis

**DOI:** 10.1007/s11695-026-08622-4

**Published:** 2026-03-23

**Authors:** Sevag Hamamah, Jamil S. Samaan, Nadine Soliman, Stephanie Nguyen, Rashmi Advani, Kamran Samakar, Kulmeet K. Sandhu, Kenneth Park, Barham K. Abu Dayyeh, Rabindra R. Watson

**Affiliations:** 1 Department of Medicine, Scripps Mercy Hospital, San Diego, USA; 2 Karsh Division of Gastroenterology and Hepatology, Cedars-Sinai Medical Center, Los Angeles, USA; 3 Faculty of Medicine, Cairo University, Giza, Egypt; 4 Division of Upper GI and General Surgery, University of Southern California, Los Angeles, USA; 5 Division of Minimally Invasive & Bariatric Surgery, Department of Surgery, Cedars-Sinai Medical Center, Los Angeles, USA

**Keywords:** Intragastric Balloon, Diabetes Mellitus, Insulin-Dependence, 30-Day Healthcare Utilization, Short-Term Outcomes

## Abstract

**Background:**

Intragastric balloon (IGB) therapy is a minimally invasive endoscopic intervention for weight reduction in patients with Class I-II obesity and in lower BMI patients with obesity-related comorbidities such as diabetes mellitus. Diabetes mellitus is associated with vascular complications, increased susceptibility to infection and delayed gastric emptying, underscoring the need to evaluate procedural safety and device tolerance in this population. However, evidence regarding short-term safety in this higher-risk population remains limited. This study aims to compare 30-day safety outcomes after IGB placement in patients with and without diabetes mellitus.

**Methods:**

This was a retrospective cohort study of 4,555 patients undergoing primary IGB placement using the Metabolic and Bariatric Surgery Accreditation and Quality Improvement Program (MBSAQIP) database from 2015 to 2023. Propensity score-matching was performed in R version 4.5.0 to balance baseline characteristics in two matched analyses: (1) patients with diabetes mellitus matched to patients without diabetes mellitus, and (2) non-insulin dependent diabetes mellitus (NIDDM) versus insulin-dependent diabetes mellitus (IDDM). Outcomes included 30-day rates of outpatient intravenous (IV) treatments, emergency department (ED) visits, hospital readmissions, reoperations, procedural interventions, and serious adverse events (SAEs).

**Results:**

Patients with diabetes, particularly those with insulin dependence, were older, more frequently male, and had a higher burden of cardiometabolic-associated medical problems compared to patients without diabetes. Among 424 propensity-matched patients with and without diabetes, rates of 30-day healthcare utilization including outpatient IV treatment, ED visit, hospital readmission, reoperation, and procedural intervention were comparable. Postoperative SAEs were rare, with no observed significant differences in rates of organ space infection, pneumonia, unplanned intubation, pulmonary embolism, deep vein thrombosis, prolonged mechanical ventilation, urinary tract infection, renal insufficiency, acute renal failure, cerebrovascular accident, cardiac arrest, myocardial infarction, sepsis, septic shock, unplanned intensive care unit admission and mortality. In a sub-analysis of 106 matched patients with NIDDM and IDDM, 30-day healthcare utilization and all postoperative SAEs were likewise similar, with no observed statistically significant differences between cohorts.

**Conclusion:**

Patients with diabetes exhibited a greater burden of associated medical problems but demonstrated comparable rates of short-term healthcare utilization, safety outcomes and tolerability to those without diabetes after adjustment for baseline differences. These findings support the safety of IGB placement in patients with diabetes and suggest it may be considered both as a safe option for primary weight loss intervention or as a bridge therapy to Metabolic and Bariatric Surgery (MBS) in this population.

**Supplementary Information:**

The online version contains supplementary material available at 10.1007/s11695-026-08622-4.

## Key points

Patients with diabetes were older, had higher BMI, and carried a greater burden of associated medical problems compared with those without diabetes.

After propensity score-matching, rates of 30-day healthcare utilization including outpatient IV treatments, emergency department visits, hospital readmissions, reoperations, procedural interventions, and postoperative serious adverse events were similar between patients with and without diabetes undergoing IGB placement.

Among patients with diabetes, those with insulin-dependent diabetes had more cardiometabolic-associated medical problems than those with non–insulin-dependent diabetes yet observed rates of 30-day healthcare utilization and postoperative serious adverse events were comparable across subgroups.

**Supplementary Information:**

The online version contains supplementary material available at 10.1007/s11695-026-08622-4.

## Introduction

Type 2 Diabetes Mellitus (T2DM), characterized by glucose dysregulation and insulin resistance, affects over 500 million individuals worldwide, representing approximately 10% of adults aged 20–75 [1, 2]. The global prevalence of T2DM is projected to rise significantly by 2050, leading to increased healthcare expenditures and mortality rates [1, 3]. Obesity, defined as a body mass index (BMI) > 30 kg/m², is the primary modifiable risk factor for T2DM due to its role in promoting insulin resistance [4]. Intragastric balloon (IGB) placement is a well-established intervention for obesity and its associated comorbidities, including T2DM [5, 6]. The mechanism of weight loss induced by IGB is multifactorial, involving gastric space occupation (≥ 400 mL), reduced meal volume, and delayed gastric emptying [5]. IGB therapy serves as a minimally invasive safe and effective intervention primarily utilized for weight loss in patients with Class I and II obesity, or in individuals with BMI 27.0–29.9 kg/m² with at least one obesity-related comorbidity, including T2DM [7].

Despite its favorable metabolic profile, the short-term procedural safety of IGB placement in patients with diabetes warrants careful evaluation. Several pathophysiological aspects of T2DM are associated with higher procedural risk including impaired wound healing, increased susceptibility to infection, and the presence of microvascular and macrovascular disease that can compromise physiologic resilience [8–11]. These effects are well-established contributors to adverse outcomes in surgical and procedural settings [12–14]. For IGB placement, another clinically relevant concern among patients with diabetes is the potential for increased device intolerance. Delayed gastric emptying is a sequela of diabetes mellitus, ranging from subtle gastric dysmotility to gastroparesis, and may exacerbate post-placement symptoms such as nausea, vomiting, abdominal discomfort, and early satiety [15]. Objective abnormalities in gastric emptying have been reported in up to 30–50% of patients with long-standing diabetes, although clinically significant gastroparesis is less common and varies by diabetes subtype [16]. These mechanisms could elevate the risk of dehydration, inability to tolerate the balloon, or early removal, thereby directly influencing short-term safety outcomes. Despite these biologically plausible risks, comparative safety data of short-term outcomes for IGB in patients with and without diabetes are limited. However, weight-loss interventions, including IGB, can improve diabetes remission rates and reduce long-term diabetes-related complications [17], further underscoring the importance of understanding short-term safety in this population.

To address this evidence gap, we analyzed 4,555 patients undergoing IGB placement in the Metabolic and Bariatric Surgery Accreditation and Quality Improvement Program (MBSAQIP) database, a nationally validated registry of 30-day perioperative and postoperative outcomes from accredited bariatric centers in the United States and Canada. We compared short-term safety outcomes between patients with and without diabetes using propensity score-matching analyses to adjust for baseline differences in comorbidities and demographic characteristics. To our knowledge, this represents the largest study to date comparing short-term safety outcomes of IGB therapy in patients with and without diabetes.

## Methods

### Data Source

We performed a retrospective cohort study from 2015 to 2023 using data from the MBSAQIP registry. The MBSAQIP is the largest bariatric-specific clinical registry in North America, collecting standardized clinical data from more than 900 accredited centers across the United States and Canada. The registry captures detailed information on patient preoperative characteristics, perioperative variables, and 30-day postoperative outcomes. All data are de-identified and compliant with the Health Insurance Portability and Accountability Act (HIPAA), and the database is subject to regular audits to ensure data accuracy and quality.

### Study Population

**Fig. 1:**
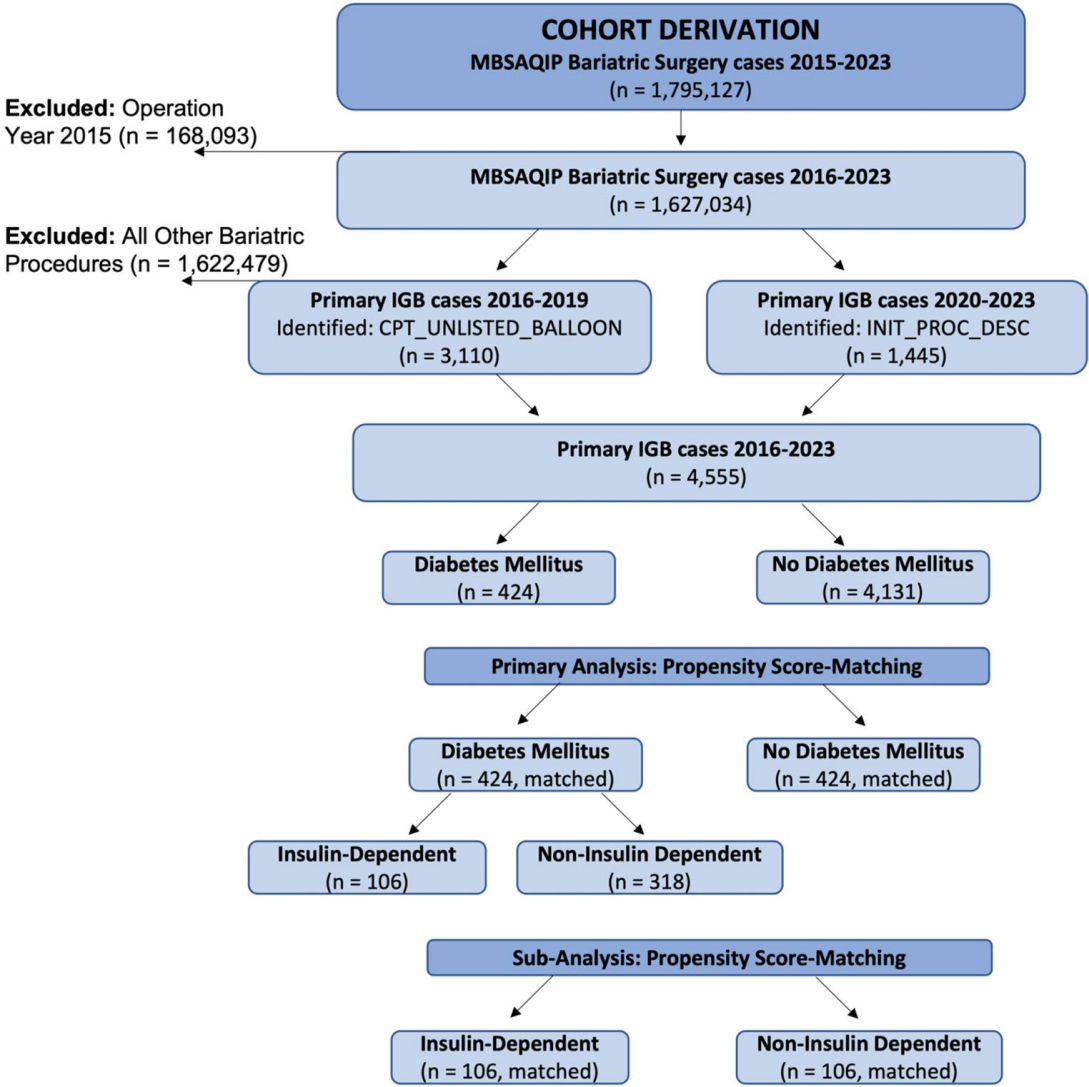
Flow Diagram of Patient Selection. Patients were identified from the MBSAQIP registry between 2015 and 2023. Cases from 2015 were excluded due to incomplete procedural classification. Primary intragastric balloon placements from 2016–2019 were identified using the CPT_UNLISTED_BALLOON variable, while cases from 2020–2023 were identified using INIT_PROC_DESC. Among 4,555 patients undergoing primary IGB placement, 424 patients with diabetes mellitus were propensity score-matched to 424 patients without diabetes. An exploratory sub-analysis was performed within the diabetic cohort comparing insulin-dependent diabetes mellitus to non-insulin-dependent diabetes mellitus, with 106 matched pairs. Abbreviations; IGB: Intragastric balloon, n: sample size

We retrospectively analyzed the MBSAQIP registry from 2015 to 2023, which contains 1,795,127 metabolic and bariatric surgeries and procedures. Cases from 2015 were excluded because no variables were available to reliably identify IGB placement. Among cases from 2016 to 2023, 4,555 patients undergoing primary IGB placement were identified, and all other procedures and surgeries, including revision cases, were excluded. Primary IGB cases from 2016 to 2019 were captured using the CPT_UNLISTED_BALLOON variable. From 2020 to 2023, identification of IGB procedures relied on the INIT_PROC_DESC variable, which provides a dedicated procedural classification for IGB placement. The variable DIABETES was used to stratify patients and included three outputs: no diabetes, non-insulin-dependent diabetes mellitus (NIDDM), and insulin-dependent diabetes mellitus (IDDM). The MBSAQIP defines an input of no diabetes as no diagnosis of diabetes or diabetes controlled by diet alone. NIDDM is defined as a diagnosis of diabetes requiring therapy with a non-insulin anti-diabetic agent, while IDDM is defined as a diagnosis of diabetes requiring daily insulin therapy. In the primary analysis, patients were stratified based on the presence or absence of diabetes mellitus and underwent propensity score-matching to create comparable cohorts. In a sub-analysis, patients with diabetes were stratified into NIDDM and IDDM and similarly underwent propensity score-matching. A flow diagram detailing patient selection and cohort derivation is shown in Fig. 1.

### Preoperative and Perioperative Variables

Demographic variables included age, body mass index (BMI), sex, and ethnicity categorized as non-Hispanic White, non-Hispanic Black, or Hispanic. Preoperative variables included recent smoking within 1 year, chronic obstructive pulmonary disease (COPD), immunosuppressant use, history of pulmonary embolism (PE), history of deep vein thrombosis (DVT), preoperative therapeutic anticoagulation use, sleep apnea, gastroesophageal reflux disease (GERD), previous foregut surgery, hypertension (HTN), hyperlipidemia (HLD), venous stasis, percutaneous coronary intervention (PCI), previous cardiac surgery, presence of inferior vena cava (IVC) filter, renal insufficiency, dialysis, independent functional status and American Society of Anesthesiologists (ASA) class > 2.

### Outcomes

The primary outcomes included procedure duration, hospital length of stay from procedure date, and 30-day rates of outpatient intravenous (IV) treatment (which includes IV treatments for nausea, vomiting, and electrolyte depletion), emergency department (ED) visits, hospital readmissions, reoperations, and procedural interventions. Primary outcomes were evaluated for the primary analysis and sub-analysis. Secondary outcomes included 30-day postoperative serious adverse events (SAEs) including organ space infection, pneumonia, unplanned intubation, PE, DVT requiring therapy, prolonged ventilation (> 48 h following the procedure), urinary tract infection (UTI), renal insufficiency, acute renal failure, cerebrovascular accident (CVA), cardiac arrest requiring cardiopulmonary resuscitation (CPR), myocardial infarction (MI), sepsis, septic shock, unplanned intensive care unit (ICU) admission and all-cause mortality.

Early balloon removal was evaluated when reliably captured within the registry. Standardized procedural descriptors allowing consistent identification of balloon removal as an intervention were introduced in later MBSAQIP registry iterations; therefore, early balloon removal was assessed in a temporal sensitivity analysis to IGB procedures performed between 2020 and 2023. Balloon removal events were identified using the INTV_TYPE variable with output intragastric balloon removal. Because balloon removal was not uniformly captured in earlier registry years (2016–2019), this outcome was not included in the primary overall analysis.

### Statistical Analysis

Categorical variables were reported as frequencies and percentages, while continuous variables were reported as means. Between-group comparisons were conducted using Student’s t-test for continuous variables and Pearson’s chi-squared or Fisher’s exact test for categorical variables, as appropriate. Chi-squared testing was used for comparisons with adequate expected cell counts, while Fisher’s exact test was applied when expected cell counts were < 5.

To account for baseline differences between groups, propensity score-matching was conducted using 1:1 nearest-neighbor matching without replacement in R version 4.5.0. DIABETES was treated as a binary exposure variable, defined as diabetes versus no diabetes in the primary analysis and NIDDM versus IDDM in the sub-analysis. Propensity scores were generated via logistic regression incorporating 19 covariates. Variables were selected a priori based on clinical relevance with perioperative risk and evidence of imbalance in the unmatched cohort. Covariate balance was evaluated using standardized mean differences (SMDs) before and after matching, with an SMD < 0.10 considered indicative of adequate post-matching balance. The overall SMD score, reflecting aggregate imbalance across all covariates, decreased from 0.735 before matching to 0.014 after propensity score-matching in the primary analysis (Supplementary Table 1), and decreased from 0.599 before matching to 0.155 after propensity score-matching in the sub-analysis (Supplementary Table 2). Because of the smaller sample size and residual confounding in the NIDDM versus IDDM comparison, this sub-analysis was considered exploratory. Following propensity score-matching, chi-squared or Fisher’s exact test were performed on matched cohorts when comparing categorical variables, and Student’s t-test for continuous variables. P-value less than 0.05 was considered statistically significant for all analyses. For continuous outcomes, mean differences (MD) with corresponding 95% confidence intervals (95%CI) were calculated. For categorical outcomes, absolute risk differences (RD) with 95%CI were reported.

A temporal sensitivity analysis was performed to evaluate potential capture bias related to changes in procedural identification variables across the study period (Supplementary Tables 3–4). Separate analyses were conducted for procedures performed between 2016 and 2019 and 2020–2023, corresponding to the use of CPT_UNLISTED_BALLOON and INIT_PROC_DESC variables, respectively. Propensity score-matching and outcome comparisons were repeated within each time period. SMD scores from these analyses are provided in Supplementary Tables 5–6. This temporal analysis was performed for the primary analysis comparing patients with and without diabetes.

Patterns of missing hemoglobin A1c (HbA1c) data were assessed using a structured missingness analysis comparing baseline demographic and clinical characteristics between patients with and without recorded HbA1c values (Supplementary Table 7). Preoperative HbA1c values were available in 512 patients (11.2%) and unavailable in 4,043 patients (88.8%). These comparisons were performed to characterize the extent and distribution of missingness data. Given the high proportion of missing HbA1c data and the limited number of patients with elevated HbA1c levels, stratified analyses by glycemic control and multiple imputation were not performed.

Given SAEs were infrequent within the matched cohorts, a detectable-difference analysis was performed to assess the study’s ability to detect between-group differences in rare binary outcomes. Using the matched cohort sizes (424 vs. 424 in the primary analysis and 106 vs. 106 in the NIDDM versus IDDM sub-analysis), we estimated the minimum absolute risk difference detectable with 80% power at an α of 0.05 for baseline event rates of 0.2%, 0.5%, 1.0%, and 2.0%. This analysis was performed to contextualize interpretation of rare SAEs and is presented in *Supplementary Table 8.*

## Results

### Primary Analysis

#### Demographics and Preoperative Comorbidities

A total of 4,555 patients underwent IGB placement, including 424 (9.3%) with diabetes and 4,131 (90.7%) without diabetes (Table 1). Patients with diabetes were older (49.6 vs. 45.5 years, *p* < 0.001), had a higher mean BMI (36.3 vs. 33.5 kg/m^2^, *p* < 0.001), and were less frequently female (69.3% vs. 83.7%, *p* < 0.001). Race and ethnicity distribution, including proportions of non-Hispanic White, non-Hispanic Black, and Hispanic patients, did not differ significantly between groups.

**Table 1:** Comparison of Demographics and Preoperative Associated Medical Problems in Patients Undergoing Intragastric Balloon (IGB) Placement, Stratified by Patients Without Diabetes versus Patients With Diabetes

Demographics	No Diabetes (*n* = 4,131)	Diabetes (*n* = 424)	*p*-value
Age (mean)	45.5	49.6	< 0.001
Body Mass Index (mean)	33.5	36.3	< 0.001
Female Sex (n, %)	3457 (83.7)	294 (69.3)	< 0.001
Non-Hispanic White (n, %)	2675 (64.8)	264 (62.3)	0.307
Non-Hispanic Black (n, %)	409 (9.9)	38 (9.0)	0.536
Hispanic Ethnicity (n, %) *	339 (9.4)	29 (7.9)	0.342

Preoperative Associated Medical Problems	No Diabetes (*n* = 4,131)	Diabetes (*n* = 424)	p-value
Smoker within One Year (n, %)	207 (5.0)	17 (4.0)	0.364
Chronic Obstructive Pulmonary Disease (n, %)	5 (0.1)	3 (0.7)	0.031
Immunosuppressant Use (n, %)	38 (0.9)	7 (1.7)	0.188
History of Pulmonary Embolism (n, %)	12 (0.3)	5 (1.2)	0.016
History of Deep Vein Thrombosis (n, %)	14 (0.3)	2 (0.5)	0.656
Therapeutic Anticoagulation Use (n, %)	24 (0.6)	13 (3.1)	< 0.001
Previous Surgery (n, %)	181 (4.4)	20 (4.7)	0.749
Sleep Apnea (n, %)	379 (9.2)	105 (24.8)	< 0.001
Gastroesophageal Reflux Disease (n, %)	927 (22.4)	142 (33.5)	< 0.001
Hypertension (n, %)	914 (22.1)	248 (58.5)	< 0.001
Hyperlipidemia (n, %)	435 (10.5)	170 (40.1)	< 0.001
Venous Stasis (n, %)	7 (0.2)	3 (0.7)	0.059
History of Myocardial Infarction (n, %)	14 (0.3)	5 (1.2)	0.026
Percutaneous Coronary Intervention (n, %)	27 (0.7)	9 (2.1)	0.005
Previous Cardiac Surgery (n, %)	21 (0.5)	5 (1.2)	0.088
Presence of Inferior Vena Cava Filter (n, %)	2 (0.0)	0 (0)	1.000
Renal Insufficiency (n, %)	6 (0.1)	3 (0.7)	0.044
Dialysis (n, %)	2 (0.0)	1 (0.2)	0.254
Independent Functional Status (n, %) *	4123 (99.9)	420 (99.1)	< 0.001
ASA Class Greater Than Two (n, %) *	3656 (94.2)	396 (98.0)	0.001

*Abbreviations:* *n *sample size,*% *percentage,*ASA *American Society of Anesthesiologists

*Denotes missing data within the covariate less than the overall sample size listed

Patients with diabetes had a higher prevalence of associated medical comorbidities, including COPD (0.7% vs. 0.1%, *p* = 0.031), history of PE (1.2% vs. 0.3%, *p* = 0.016), preoperative therapeutic anticoagulation use (3.1% vs. 0.6%, *p* < 0.001), sleep apnea (24.8% vs. 9.2%, *p* < 0.001), GERD (33.5% vs. 22.4%, *p* < 0.001), HTN (58.5% vs. 22.1%, *p* < 0.001), HLD (40.1% vs. 10.5%, *p* < 0.001), history of MI (1.2% vs. 0.3%, *p* = 0.026), prior PCI (2.1% vs. 0.7%, *p* = 0.005), renal insufficiency (0.7% vs. 0.1%, *p* = 0.044), and ASA class > 2 (98.0% vs. 94.2%, *p* = 0.001). The percentage of patients with independent functional status was lower in individuals with diabetes (99.1% vs. 99.9%, *p* < 0.001). There were no statistically significant differences in recent smoking, immunosuppressant use, history of DVT, previous surgery, venous stasis, prior cardiac surgery, IVC filter placement, or dialysis dependence (all *p* > 0.05).

### Perioperative Factors, 30-Day Healthcare Utilization and Postoperative Serious Adverse Events

In the propensity score-matched cohort (424 patients with diabetes and 424 without diabetes), perioperative factors and 30-day postoperative outcomes were overall comparable between groups (Table 2). Mean procedure length (16.8 vs. 15.9 min; MD 0.9, 95%CI -1.2 to 2.9; *p* = 0.425) and rates of hospital stay of greater than one day following the procedure (4.5% vs. 2.6%; RD 1.9%, 95%CI -0.6% to 4.4%; *p* = 0.137) were similar.

**Table 2:** Procedure Length and Postoperative 30-day Healthcare Utilization Following Intragastric Balloon (IGB) Placement in Patients Without Diabetes versus With Diabetes Within Propensity Matched Cohorts

Perioperative Parameters	No Diabetes (*n* = 424)	Diabetes (*n* = 424)	*p*-value	MD (95CI)
Procedure Length in Minutes (mean)	15.9	16.8	0.425	0.9 (-1.2, 2.9)

30-Day Healthcare Utilization	No Diabetes (*n* = 424)	Diabetes (*n* = 424)	p-value	RD% (95CI)
Outpatient Intravenous Treatment (n, %)	25 (5.9)	31 (7.3)	0.407	1.4 (-1.9, 4.8)
Greater Than One Day from Procedure to Discharge (n, %)	11 (2.6)	19 (4.5)	0.137	1.9 (-0.6, 4.4)
Emergency Department Visit (n, %)	22 (5.1)	22 (5.1)	1.000	0.0 (-3.0, 3.0)
Readmission (n, %)	12 (2.8)	18 (4.2)	0.265	1.4 (-1.1, 3.9)
Reoperation (n, %)	6 (1.4)	5 (1.2)	0.762	0.2 (-1.7, 2.3)
Intervention (n, %)	25 (5.9)	27 (6.4)	0.775	0.5 (-2.8, 3.7)

*Abbreviations:* *n* sample Size, *%* Percentage, *MD* Mean Difference, *RD%* Risk Difference Percentage, *95CI* 95% Confidence Interval

There was no statistically significant difference in 30-day healthcare utilization, including rates of outpatient IV treatment (7.3% vs. 5.9%; RD 1.4%, 95%CI -1.9% to 4.8%; *p* = 0.407), ED visit (5.1% vs. 5.1%; RD 0.0%, 95%CI -3.0% to 3.0%; *p* = 1.000), hospital readmission (4.2% vs. 2.8%; RD 1.4%, 95%CI -1.1% to 3.9%; *p* = 0.265), reoperation (1.2% vs. 1.4%; RD 0.2%, 95%CI -1.7% to 2.3%; *p* = 0.762), and procedural intervention (6.4% vs. 5.9%; RD 0.5%, 95%CI -2.8% to 3.7%; *p* = 0.775).

30-day postoperative outcomes were rare in both cohorts (Table 3). There was no organ space infection, PE, DVT requiring therapy, prolonged ventilation greater than 48 h, UTI, renal insufficiency, acute renal failure, CVA, sepsis, or septic shock in either cohort. The rates of pneumonia, cardiac arrest requiring CPR, unplanned intubations, myocardial infarctions, unplanned ICU admissions and mortality were low in both cohorts and did not reach statistical significance.

**Table 3:** Postoperative 30-day Serious Adverse Events (SAEs) Following Intragastric Balloon (IGB) Placement in Patients Without Diabetes versus With Diabetes Within Propensity Matched Cohorts

30-Day Postoperative Serious Adverse Events	No Diabetes (*n* = 424)	Diabetes (*n* = 424)	*p*-value	RD% (95CI)
Organ Space Infection (n, %)	0 (0)	0 (0)	--	--
Pneumonia (n, %)	1 (0.2)	1 (0.2)	1.000	0.0 (-2.3, 2.3)
Unplanned Intubation (n, %)	2 (0.5)	1 (0.2)	1.000	-0.3 (-1.0, 0.6)
Pulmonary Embolism (n, %)	0 (0)	0 (0)	--	--
Deep Vein Thrombosis (n, %)	0 (0)	0 (0)	--	--
On-Ventilator > 48 h (n, %)	0 (0)	0 (0)	--	--
Urinary Tract Infection (n, %)	0 (0)	0 (0)	--	--
Renal Insufficiency (n, %)	0 (0)	0 (0)	--	--
Acute Renal Failure (n, %)	0 (0)	0 (0)	--	--
Cerebrovascular Accident (n, %)	0 (0)	0 (0)	--	--
Cardiac Arrest Requiring CPR (n, %)	1 (0.2)	1 (0.2)	1.000	0.0 (-2.3, 2.3)
Myocardial Infarction (n, %)	0 (0)	1 (0.2)	1.000	0.2 (-0.2, 0.7)
Sepsis (n, %)	0 (0)	0 (0)	--	--
Septic Shock (n, %)	0 (0)	0 (0)	--	--
Unplanned ICU Admission (n, %)	1 (0.2)	2 (0.5)	1.000	0.3 (-0.6, 1.0)
Mortality (n, %)	1 (0.2)	1 (0.2)	1.000	0.0 (-2.3, 2.3)

*Abbreviations:* *n* Sample Size, *%* Percentage, -- Not Available, > Greater Than, *CPR* Cardiopulmonary Resuscitation, *ICU* Intensive Care Unit, *RD%* Risk Difference Percentage, *95CI* 95% Confidence Interval

Temporal sensitivity analyses demonstrated findings consistent with the primary analysis. In the propensity score-matched cohort restricted to procedures performed between 2016 and 2019, perioperative parameters, healthcare utilization, and 30-day postoperative complications were comparable between patients with and without diabetes (Supplementary Table 3). Similarly, in the 2020–2023 cohort, there were no significant differences in perioperative factors, healthcare utilization, or postoperative complications between groups (Supplementary Table 4). Rates of early balloon removal were also not statistically significant between patients with and without diabetes (6.3% vs. 4.5%; RD 1.8%, 95%CI -4.1% to 7.7%; *p* = 0.768).

### Sub-analysis

#### Demographics and Preoperative Comorbidities

Among 424 patients with diabetes who underwent IGB placement, 318 (75.0%) had NIDDM and 106 (25.0%) had IDDM (Table 4). Patients with IDDM were older (52.4 vs. 48.7 years, *p* = 0.004) and less frequently female (57.5% vs. 73.3%, *p* = 0.002). BMI and race/ethnicity distribution, including proportions of non-Hispanic White, non-Hispanic Black, and Hispanic patients, were similar between groups.

**Table 4:** Comparison of Demographics and Preoperative Associated Medical Problems in Patients Undergoing Intragastric Balloon (IGB) Placement, Stratified by Patients with Non-Insulin Dependent Diabetes Mellitus (NIDDM) versus Insulin-Dependent Diabetes Mellitus (IDDM)

Demographics	NIDDM (*n* = 318)	IDDM (*n* = 106)	*p*-value
Age (mean)	48.7	52.4	0.004
Body Mass Index (mean)	36.0	37.3	0.328
Female Sex (n, %)	233 (73.3)	61 (57.5)	0.002
Non-Hispanic White (n, %)	197 (61.9)	67 (63.2)	0.817
Non-Hispanic Black (n, %)	27 (8.5)	11 (10.4)	0.556
Hispanic Ethnicity (n, %) *	22 (8.0)	7 (7.5)	0.884

Preoperative Associated Medical Problems	NIDDM (*n* = 318)	IDDM (*n* = 106)	p-value
Smoker within One Year (n, %)	12 (3.8)	5 (4.7)	0.775
Chronic Obstructive Pulmonary Disease (n, %)	1 (0.3)	2 (1.9)	0.156
Immunosuppressant Use (n, %)	4 (1.3)	3 (2.8)	0.374
History of Pulmonary Embolism (n, %)	2 (0.6)	3 (2.8)	0.102
History Deep Vein Thrombosis (n, %)	0 (0)	2 (1.9)	0.062
Therapeutic Anticoagulation Use (n, %)	5 (1.6)	8 (7.5)	0.005
Previous Surgery (n, %)	16 (5.0)	4 (3.8)	0.597
Sleep Apnea (n, %)	75 (23.6)	30 (28.3)	0.330
Gastroesophageal Reflux Disease (n, %)	112 (35.2)	30 (28.3)	0.191
Hypertension (n, %)	169 (53.1)	79 (74.5)	< 0.001
Hyperlipidemia (n, %)	111 (34.9)	59 (55.7)	< 0.001
Venous Stasis (n, %)	2 (0.6)	1 (0.9)	1.000
History of Myocardial Infarction (n, %)	2 (0.6)	3 (2.8)	0.102
Percutaneous Coronary Intervention (n, %)	3 (0.9)	6 (5.7)	0.009
Previous Cardiac Surgery (n, %)	1 (0.3)	4 (3.8)	0.015
Presence of Inferior Vena Cava Filter (n, %)	0 (0)	0 (0)	--
Renal Insufficiency (n, %)	3 (0.9)	0 (0)	0.576
Dialysis (n, %)	0 (0)	1 (0.9)	0.250
Independent Functional Status * (n, %)	315 (99.1)	105 (99.1)	1.000
ASA Class Greater Than Two * (n, %)	298 (98.0)	98 (98.0)	1.000

*Abbreviations:* *NIDDM* Non-insulin Dependent Diabetes Mellitus, *IDDM* Insulin-dependent Diabetes Mellitus, *n* Sample Size, *%* Percentage, *ASA* American Society of Anesthesiologists, -- Not Applicable

*Denotes missing data within the covariate less than the overall sample size listed

Patients with IDDM demonstrated higher rates of preoperative associated medical problems compared with those with NIDDM. These included greater use of preoperative therapeutic anticoagulation use (7.5% vs. 1.6%, *p* = 0.005), HTN (74.5% vs. 53.1%, *p* < 0.001), HLD (55.7% vs. 34.9%, *p* < 0.001), prior PCI (5.7% vs. 0.9%, *p* = 0.009), and previous cardiac surgery (3.8% vs. 0.3%, *p* = 0.015). There were no statistically significant differences in rates of recent smoking, COPD, immunosuppressant use, history of PE, history of DVT, previous surgery, sleep apnea, GERD, venous stasis, history of MI, renal insufficiency, dialysis, functional status, and ASA class (all *p* > 0.05).

### Perioperative Factors, 30-Day Healthcare Utilization and Postoperative Serious Adverse Events

In the propensity score–matched cohort (106 patients with NIDDM and 106 with IDDM), perioperative factors and 30-day postoperative outcomes were similar (Table 5). Given the relatively small, matched cohort size and residual covariate imbalance, the NIDDM versus IDDM comparison should be interpreted as an exploratory sub-analysis. Mean procedure length (17.3 vs. 16.2 min; MD 1.1, 95%CI -3.8 to 6.0; *p* = 0.651) and rates of a post-procedural hospital stay of greater than one day (5.7% vs. 2.8%; RD 2.9%, 95%CI -2.6% to 8.2%; *p* = 0.498) were comparable.

**Table 5:** Procedural Length and Postoperative 30-day Healthcare Utilization Following Intragastric Balloon (IGB) Placement in Patients with Non-Insulin Dependent Diabetes Mellitus (NIDDM) versus Insulin-Dependent Diabetes Mellitus (IDDM) Within Propensity Matched Cohorts

Perioperative Parameters	NIDDM (*n* = 106)	IDDM (*n* = 106)	*p*-value	MD (95CI)
Procedure Length in Minutes (mean)	16.2	17.3	0.651	1.1 (-3.8, 6.0)

30-Day Healthcare Utilization	NIDDM (*n* = 106)	IDDM (*n* = 106)	*p*-value	RD% (95CI)
Outpatient Intravenous Treatment (n, %)	11 (10.4)	8 (7.5)	0.471	-2.9 (-10.5, 4.9)
Greater Than One Day from Procedure to Discharge (n, %)	3 (2.8)	6 (5.7)	0.498	2.9 (-2.6, 8.2)
Emergency Department Visit (n, %)	6 (5.7)	8 (7.5)	0.580	1.8 (-4.8, 8.6)
Readmission (n, %)	8 (7.5)	7 (6.6)	0.789	-0.9 (-7.8, 6.0)
Reoperation (n, %)	1 (1.0)	2 (1.9)	1.000	0.9 (-2.0, 4.1)
Intervention (n, %)	11 (10.3)	10 (9.4)	0.818	-0.9 (-9.0, 7.1)

*Abbreviations:NIDDM* Non-insulin Dependent Diabetes Mellitus, *IDDM* Insulin-dependent Diabetes Mellitus, *n* Sample Size, % Percentage, *MD* Mean Difference, *RD%* Risk Difference Percentage, *95CI* 95% Confidence Interval

Rates of 30-day healthcare utilization, including outpatient IV treatment (7.5% vs. 10.4%; RD -2.9%, 95%CI -10.5% to 4.9%; *p* = 0.471), ED visit (7.5% vs. 5.7%; RD 1.8%, 95%CI -4.8% to 8.6%; *p* = 0.580), hospital readmission (6.6% vs. 7.5%; RD -0.9%, 95%CI -7.8% to 6.0%; *p* = 0.789), reoperation (1.9% vs. 1.0%; RD 0.9%, 95%CI -2.0% to 4.1%; *p* = 1.000) and procedural intervention (9.4% vs. 10.3%; RD -0.9%, 95%CI -9.0% to 7.1%; *p* = 0.818) did not differ significantly between NIDDM and IDDM patients. 30-day postoperative SAEs were rare in both cohorts (Table 6). There was no organ space infection, PE, DVT requiring therapy, prolonged ventilation greater than 48 h, UTI, renal insufficiency, acute renal failure, CVA, cardiac arrest requiring CPR, sepsis, or septic shock in both cohorts. There were no deaths in either group within 30 days of the procedure. The rates of pneumonia, unplanned intubation, myocardial infarction, and unplanned ICU admission were low or zero in both cohorts and did not reach statistical significance.

**Table 6:** Postoperative 30-Day Serious Adverse Events (SAEs) Following Intragastric Balloon (IGB) Placement in Patients with Non-Insulin Dependent Diabetes Mellitus (NIDDM) versus Insulin-Dependent Diabetes Mellitus (IDDM) Within Propensity Matched Cohorts

30-Day Postoperative Serious Adverse Events	NIDDM (*n* = 106)	IDDM (*n* = 106)	*p*-value	RD% (95CI)
Organ Space Infection (n, %)	0 (0)	0 (0)	--	--
Pneumonia (n, %)	1 (1.0)	0 (0)	1.000	-1.0 (-2.8, 0.9)
Unplanned Intubation (n, %)	1 (1.0)	0 (0)	1.000	-1.0 (-2.8, 0.9)
Pulmonary Embolism (n, %)	0 (0)	0 (0)	--	--
Deep Vein Thrombosis (n, %)	0 (0)	0 (0)	--	--
On-Ventilator > 48 h (n, %)	0 (0)	0 (0)	--	--
Urinary Tract Infection (n, %)	0 (0)	0 (0)	--	--
Renal Insufficiency (n, %)	0 (0)	0 (0)	--	--
Acute Renal Failure (n, %)	0 (0)	0 (0)	--	--
Cerebrovascular Accident (n, %)	0 (0)	0 (0)	--	--
Cardiac Arrest Requiring CPR (n, %)	0 (0)	0 (0)	--	--
Myocardial Infarction (n, %)	0 (0)	1 (1.0)	1.000	1.0 (-0.9, 2.8)
Sepsis (n, %)	0 (0)	0 (0)	--	--
Septic Shock (n, %)	0 (0)	0 (0)	--	--
Unplanned ICU Admission (n, %)	1 (1.0)	0 (0)	1.000	-1.0 (-2.8, 0.9)
Mortality (n, %)	0 (0)	0 (0)	--	--

*Abbreviations*: *NIDDM* Non-insulin Dependent Diabetes Mellitus, *IDDM* Insulin-dependent Diabetes Mellitus, *n* Sample Size, % Percentage -- Not Applicable, > Greater Than, *CPR* Cardiopulmonary Resuscitation, *ICU* Intensive Care Unit, *RD%* Risk Difference Percentage, *95CI* 95% Confidence Interval

## Discussion

As IGB therapy is increasingly incorporated into obesity management for patients with diabetes mellitus, its short-term safety merits careful evaluation given diabetes-associated delayed gastric emptying and autonomic dysfunction that may predispose to early device intolerance. These mechanisms raise the potential for increased nausea, vomiting, and dehydration following placement of space-occupying gastric devices, making evaluation of clinically meaningful short-term outcomes essential.

In this large analysis of the MBSAQIP database, patients with diabetes undergoing IGB placement did not experience higher rates of 30-day healthcare utilization and intolerance-related outcomes compared with patients without diabetes. After propensity score-matching, primary 30-day outcomes, including hospital length of stay, outpatient IV treatments, ED visits, readmissions, reoperations, and procedural interventions were comparable between groups. Similarly, in our sensitivity analysis of years 2020 to 2023, no significant differences in balloon removal were observed. The absence of increased healthcare utilization and intolerance outcomes after adjustment suggests that diabetes itself may not confer excess short-term procedural risk following IGB placement. Within the diabetic cohort, primary outcomes remained comparable between patients with IDDM and NIDDM after matching. Across both analyses, 30-day postoperative SAEs were rare, and rates were comparable. Collectively, these findings suggest that IGB therapy does not appear to be associated with higher observed rates of short-term healthcare utilization, SAEs or intolerability in patients with diabetes, including those with insulin dependence.

Current guidelines from the American Society for Gastrointestinal Endoscopy (ASGE), European Society of Gastrointestinal Endoscopy (ESGE) and the American Gastroenterological Association (AGA) support IGB placement as a primary endoscopic therapy for select patients with BMI ≥ 30 kg/m^2^ or patients with BMI 27.0–29.9 kg/m^2^ with at least one obesity-related comorbidity (e.g. T2DM) [18]. Pooled data mentioned in these guidelines from 5 randomized control trials (RCTs) and 18 observational trials found that IGB lowered HbA1c levels more than noninvasive therapy, especially in patients with baseline HbA1c > 6.5% and in patients with a BMI > 40 kg/m^2^ [19]. While the metabolic efficacy of IGB in patients with diabetes is well-established, high-quality data specifically evaluating the short-term procedural safety in this population have been comparatively limited. Regarding safety, pooled data from seven RCTs cited in the guidelines estimate an absolute risk of 32 SAEs per 1,000 patients undergoing IGB placement compared to controls. However, these analyses were not conducted specifically in patients with diabetes, leaving uncertainty regarding procedural safety in this population [18]. Our findings help address this gap by demonstrating reassuring early safety outcomes in a large, nationally representative cohort.

The guidelines further support IGB as a bridge therapy for high-risk surgical candidates [18]. Individuals with advanced diabetes, severe obesity, or significant cardiometabolic disease may face elevated perioperative risk when undergoing surgical bariatric procedures [20, 21]. Preoperative weight reduction has been shown to improve metabolic parameters [22] and facilitate safer subsequent bariatric surgery by improving operative exposure and reducing surgical complexity [23, 24]. This bridging strategy may be particularly relevant for patients with poorly controlled diabetes, in whom optimization of metabolic status prior to surgery can reduce perioperative morbidity. Prior MBSAQIP data have shown higher 30-day mortality and SAEs after laparoscopic sleeve gastrectomy (LSG), and a higher risk of SAEs after laparoscopic Roux-en-Y Gastric Bypass (L-RYGB) in patients with IDDM compared to those without [13], highlighting the heightened perioperative vulnerability of populations with advanced diabetes. In contrast, our study did not identify statistically significant differences in short-term safety outcomes between patients with and without insulin dependence undergoing IGB placement. This distinction underscores the potential role of IGB therapy as a minimally invasive, anatomy-sparing therapeutic option that may aid in optimizing metabolic control and physiologic status prior to definitive bariatric surgery in appropriately selected patients with diabetes.

Previous large-scale studies and meta-analyses have reported significant improvements in glycemic control following IGB placement, with low overall rates of SAEs, but have not directly compared outcomes between diabetic and non-diabetic cohorts. A systematic review reported significant reductions in HbA1c six months after IGB placement without systematic evaluation of SAEs [22], while a subsequent meta-analysis of randomized and observational studies reported a pooled SAE rate of 1.3% [25]. Additional prospective and retrospective studies have similarly reported improvements in HbA1c following IGB therapy in patients with T2DM [26] and low rates of major and minor complications (0.5% and 5.4%, respectively) [27]. These findings are consistent with the low adverse event rates in diabetic cohorts observed in our study. However, the absence of propensity-matched comparisons and direct diabetic vs. non-diabetic analyses in prior studies limits their ability to isolate the impact of diabetes on procedural safety. Our study addresses this limitation by providing a matched, comparative assessment of short-term outcomes.

While metabolic efficacy is an important consideration in evaluating IGB therapy for patients with diabetes, the present analysis was not designed to assess weight loss or glycemic improvement. The MBSAQIP registry is structured to capture perioperative characteristics and standardized 30-day postoperative outcomes rather than longitudinal metabolic efficacy. Although postoperative BMI values are recorded within the dataset, the timing of these measurements varies considerably across patients and does not represent a uniform follow-up interval. As a result, reliable estimation of short-term weight loss metrics such as percent total body weight loss is difficult to address with the available data. Additionally, postoperative HbA1c values are not captured within the registry, precluding assessment of glycemic improvement or diabetes remission. Consequently, the present study focuses specifically on short-term safety and intolerance-related outcomes, which are systematically and reliably captured within the MBSAQIP database.

A clinically important finding of our study is the comparable rate of outpatient IV treatment for dehydration between cohorts. As mentioned, diabetes is associated with delayed gastric emptying and autonomic dysfunction, raising concern for increased gastrointestinal intolerance symptoms following placement of space-occupying gastric devices. Given that many intolerance symptoms may be managed in outpatient settings without prompting ED visits or hospital readmission, we sought to capture clinically relevant intolerance to the fullest extent possible using available MBSAQIP variables. Outpatient IV treatments for nausea, vomiting or electrolyte depletion represent the most pragmatic surrogate available within the MBSAQIP database for clinically meaningful intolerance that did not lead to hospital-based encounters. The lack of a significant difference in this outcome suggests that diabetes, including insulin dependence, does not appear to confer an increased risk of early intolerance requiring outpatient intervention, supporting the overall tolerability of IGB therapy in this population. Similarly, early balloon removal represents a clinically meaningful indicator of device intolerance, particularly in patients with diabetes mellitus. Although no significant differences were observed in the 2020–2023 cohort, evaluation across the entire study period was limited because this outcome could only be reliably captured in later iterations of the registry.

The absence of detailed anti-hyperglycemic medication data represents a clinically meaningful limitation with direct implications for interpretation of the observed findings of our study. During the study period, the use of glucagon-like peptide-1 receptor agonists (GLP-1 RAs), including liraglutide and semaglutide, expanded substantially among patients with obesity and T2DM [28, 29]. GLP-1 RAs are known to delay gastric emptying and increase gastrointestinal adverse effects, including nausea and vomiting, which may be additive to the space-occupying effects of IGB therapy [30]. Concurrent use of GLP-1 RA could therefore plausibly increase intolerance risk following balloon placement, an effect that cannot be evaluated within the current dataset.

Similarly, sodium-glucose cotransporter-2 (SGLT-2) inhibitors have been associated with an increased risk of perioperative euglycemic diabetic ketoacidosis (DKA), particularly in the setting of reduced oral intake or physiologic stress [31, 32]. The inability to capture medication exposure precludes assessment of medication-related metabolic complications that may influence early postoperative outcomes, including complications not captured by the database such as DKA. Accordingly, the reassuring safety profile observed in this study should be interpreted in the context of unmeasured medications effects, and future prospective studies should strongly consider capture of anti-hyperglycemic medication data to enable comprehensive procedural risk assessment.

IGB systems used during the study period likely differed in fill medium, weight, and gastric distension characteristics, factors that may influence gastrointestinal tolerability and gastric motility [33]. Fluid-filled balloons, which represented the predominant device used during a majority of the study period following United States regulatory approvals in 2015 [6, 34], exert greater intragastric mass and mechanical distension than gas-filled balloons [35]. In contrast, gas-filled systems are substantially lighter and have been associated with improved gastrointestinal tolerability compared with fluid-filled balloons [36], although weight loss outcomes may be modestly lower [35]. Adjustable fluid-filled systems, introduced later in the study period, allow endoscopic modification of balloon volume to mitigate intolerance, but exert similar physiologic effects on gastric emptying and distension [37]. These physiologic differences are relevant in patients with diabetes-related gastric dysmotility. In this context, the greater intragastric mass of fluid-filled balloons may further impair gastric emptying in patients with autonomic neuropathy, whereas lighter gas-filled systems may exert less mechanical influence of gastric motility and therefore be associated with improved gastrointestinal tolerability. However, device-level analyses could not be reliably performed within the MBSAQIP registry across the study period. However, a temporal sensitivity analysis 2016 to 2019 and 2020 to 2023 did not demonstrate significant differences in intolerance-related outcomes, suggesting that differences in device adoption over time were unlikely to meaningfully influence the primary findings.

The interpretation of outcomes within the IDDM subgroup warrants careful qualification. The MBSAQIP variable for insulin-dependent diabetes mellitus does not distinguish between type 1 diabetes mellitus (T1DM) and T2DM, two entities with fundamentally distinct pathophysiology, disease duration, autonomic neuropathy burden, and prevalence of gastroparesis [16, 38]. As a result, the IDDM cohort likely represents a pathophysiologically heterogeneous population, limiting the ability to generalize subgroup findings specifically to either T1DM or insulin-treated T2DM. Differences in gastric neuromuscular dysfunction and autonomic impairment, comparatively more pronounced in long-standing T1DM [39], may differentially influence intolerance risk following space-occupying gastric devices. Consequently, while no excess short-term risk was observed within the aggregated IDDM cohort, these findings should not be interpreted as definitive evidence of equivalent safety across distinct insulin-dependent diabetes phenotypes. Future prospective studies should explicitly differentiate diabetes subtype to permit mechanistically meaningful risk stratification. Additionally, the NIDDM versus IDDM comparison should be interpreted as exploratory. Although propensity score-matching substantially reduced baseline imbalance, complete covariate balance in the sub-analysis was not achieved (post-matching overall SMD = 0.155), likely reflecting the limited sample size of the matched cohorts and inherent clinical differences between diabetes subtypes. Consequently, residual confounding cannot be excluded.

This study has several additional limitations that warrant consideration. First, clinical management decisions, including ED evaluation, readmissions, reoperations, and interventions were made at the discretion of treating providers, which may contribute to inter-institutional variability. Heterogeneity in local protocols, documentation practices, and coding accuracy may also influence reliability of registry-based data, including the MBSAQIP. Standardized symptom reporting is also absent, therefore, milder gastrointestinal intolerance symptoms managed conservatively in the outpatient setting without IV treatments are likely underestimated. Assessment of diabetes severity was limited by frequently missing preoperative HbA1c data, which were unavailable in 88.8% of patients undergoing IGB placement in this cohort, precluding stratification by baseline glycemic control and evaluation of its association with outcomes. Analysis of missingness patterns demonstrated that patients with recorded HbA1c values had higher mean body mass index and a greater prevalence of metabolic comorbidities compared with those without recorded HbA1c values. These findings suggest non-random missingness of HbA1c data within the MBSAQIP registry, likely reflecting institutional documentation practices and patient selection. Given the extent of missing data and the small number of patients with elevated HbA1c values, incorporation of HbA1c into analytic models or stratification by glycemic control was not methodologically appropriate. Consequently, diabetes severity could not be reliably assessed, limiting metabolic contextualization. Insulin dependence was therefore used as a pragmatic surrogate for disease severity, with inherent limitations described earlier.

The retrospective nature of the study limits ability to establish causality and residual confounding cannot be fully excluded despite propensity score-matching. Selection bias remains possible, as factors influencing candidacy for IGB placement are incompletely captured within the dataset. Lastly, interpretation of the findings requires consideration of the low absolute event rates of 30-day healthcare utilization and postoperative SAEs. While the matched cohorts were sufficiently large to evaluate moderate differences between cohorts, the rarity of adverse outcomes limits the ability to discern smaller distinctions related to diabetes and insulin dependence. Detectable-difference analysis demonstrated that, with 424 matched patients per group, absolute differences of approximately 2–4% would be required to detect statistical significance for rare events occurring at rates ≤ 2%. In the NIDDM versus IDDM matched sub-analysis with 106 matched pairs, detectable differences were substantially larger (approximately 7–10%), reflecting the smaller sample size of this exploratory comparison. Therefore, smaller absolute differences in rare serious adverse events occurring at rates below 1% would not be expected to reach statistical significance within the available sample size. Therefore, modest variations in primary outcomes cannot be excluded, and the absence of statistically significant differences in rare SAEs should not be interpreted as evidence of clinical equivalence or non-inferiority between cohorts. Generalizability may also be limited, as MBSAQIP primarily includes accredited bariatric centers, which may differ from community practices where IGB is increasingly offered. Despite these constraints, this remains the largest comparative study of IGB outcomes in patients with and without diabetes.

## Conclusion

Preoperative diabetes mellitus, including IDDM, was not associated with increased 30-day postoperative SAEs or healthcare utilization following IGB placement in this large, multicenter cohort. After adjustment for baseline differences in comorbidity burden, short-term safety outcomes and healthcare utilization were comparable between patients with and without diabetes. These findings support the role of IGB placement as a minimally invasive option within individualized obesity treatment pathways for patients with diabetes. Prospective studies with longer follow-up and rigorous methodologic design are warranted to evaluate durability of metabolic benefit, long-term safety, and patient-centered outcomes in this population.

## Supplementary Information

Below is the link to the electronic supplementary material.

Supplementary Material 1.

## Data Availability

Data is available to participating centers upon request from the Metabolic and Bariatric Surgery Accreditation and Quality Improvement Program (MBSAQIP).
