# Supplementary material for: Short-Term Safety and Healthcare Utilization Following Intragastric Balloon Placement in Patients With and Without Diabetes Mellitus: A Propensity-Matched Analysis
Source: Obes Surg. 2026 Mar 23;36(4):1849–61. doi: 10.1007/s11695-026-08622-4 (PMC13083371; doi:10.1007/s11695-026-08622-4)
Supplement: Supplementary file 1 — Supplementary Material 1. [file 11695_2026_8622_MOESM1_ESM.docx]

***Supplementary Table 1.*** Standard Mean Differences After Propensity Score Matching of 19 Matched Covariates for Analysis Comparing Cohorts with and without Diabetes Undergoing Intragastric Balloon (IGB) Placement.

| **Covariate** | **Standard Mean Difference** |
| --- | --- |
| Female Sex | 0.010 |
| Age Greater than 45 | 0.015 |
| Non-Hispanic White | 0.069 |
| Body Mass Index Greater than 35 | 0.083 |
| Smoker within One Year | 0.065 |
| Immunosuppressant Use | 0.018 |
| Chronic Obstructive Pulmonary Disease | < 0.001 |
| History of Pulmonary Embolism | 0.023 |
| Sleep Apnea | 0.054 |
| Gastroesophageal Reflux Disease | 0.020 |
| Previous Surgery | 0.035 |
| History of Myocardial Infarction | 0.049 |
| Previous Percutaneous Coronary Intervention | 0.074 |
| Hypertension | 0.010 |
| Hyperlipidemia | < 0.001 |
| History of Deep Vein Thrombosis | 0.031 |
| Venous Stasis | 0.119 |
| Therapeutic Anticoagulation Use | 0.059 |
| Renal Insufficiency | < 0.001 |
| Overall Match Distance | |
| Pre-Match Distance | Post-Match Distance |
| 0.735 | 0.014 |

***Supplementary Table 2*.** Standard Mean Differences After Propensity Score Matching of 19 Matched Covariates for Analysis Comparing Cohorts with Non-Insulin Dependent Diabetes Mellitus (NIDDM) versus Insulin-Dependent Diabetes Mellitus (IDDM) Undergoing Intragastric Balloon (IGB) Placement.

| **Covariate** | **Standard Mean Difference** |
| --- | --- |
| Female Sex | 0.115 |
| Age Greater than 45 | 0.086 |
| Non-Hispanic White | 0.202 |
| Body Mass Index Greater than 35 | 0.172 |
| Smoker within One Year | < 0.001 |
| Immunosuppressant Use | 0.062 |
| Chronic Obstructive Pulmonary Disease | 0.080 |
| History of Pulmonary Embolism | 0.138 |
| Sleep Apnea | 0.107 |
| Gastroesophageal Reflux Disease | 0.042 |
| Previous Surgery | < 0.001 |
| History of Myocardial Infarction | 0.138 |
| Prior Percutaneous Coronary Intervention | 0.140 |
| Hypertension | 0.022 |
| Hyperlipidemia | 0.019 |
| History of Deep Vein Thrombosis | 0.195 |
| Venous Stasis | 0.137 |
| Therapeutic Anticoagulation Use | 0.118 |
| Renal Insufficiency | < 0.001 |
| Overall Match Distance | |
| Pre-Match Distance | Post-Match Distance |
| 0.599 | 0.155 |

***Supplementary Table 3.*** Temporal Sensitivity Analysis of 30-Day Postoperative Outcomes Following Intragastric Balloon (IGB) Placement (2016-2019 Cohort), Stratified by Diabetes Status After Propensity Score Matching.

| **Perioperative Parameters** | **No Diabetes (n = 313)** | **Diabetes (n = 313)** | **p-value** | **MD (95CI)** |
| --- | --- | --- | --- | --- |
| Procedure Length (mean) | 17.5 | 18.4 | 0.533 | 0.9 (-1.9, 3.7) |
| **30-Day Healthcare Utilization** | **No Diabetes (n = 313)** | **Diabetes (n = 313)** | **p-value** | **RD% (95CI)** |
| Outpatient Intravenous Treatment (n, %) | 21 (6.7) | 23 (7.3) | 0.755 | 0.6 (-3.3, 6.7) |
| Greater Than One Day From Procedure to Discharge (n, %) | 12 (3.8) | 16 (5.1) | 0.439 | 1.3 (-2.0, 4.5) |
| Emergency Department Visit (n, %) | 17 (5.4) | 12 (3.8) | 0.342 | -1.6 (-4.9, 1.7) |
| Readmission (n, %) | 9 (2.9) | 12 (3.8) | 0.657 | 0.9 (-1.8, 3.7) |
| Reoperation (n, %) | 5 (1.6) | 4 (1.3) | 1.000 | -0.3 (-2.1, 1.5) |
| Intervention (n, %) | 31 (9.9) | 20 (6.4) | 0.108 | -3.5 (-7.8, 0.8) |
| **30-Day Postoperative Serious Adverse Events** | **No Diabetes (n = 313)** | **Diabetes (n = 313)** | **p-value** | **RD% (95CI)** |
| Organ Space Infection (n, %) | 0 (0) | 0 (0) | -- | -- |
| Pneumonia (n, %) | 1 (0.3) | 1 (0.3) | 1.000 | 0 (-0.9, 0.9) |
| Unplanned Intubation (n, %) | 1 (0.3) | 1 (0.3) | 1.000 | 0 (-0.9, 0.9) |
| Pulmonary Embolism (n, %) | 0 (0) | 0 (0) | -- | -- |
| Deep Vein Thrombosis (n, %) | 0 (0) | 0 (0) | -- | -- |
| On Ventilator > 48 hours (n, %) | 0 (0) | 0 (0) | -- | -- |
| Urinary Tract Infection (n, %) | 1 (0.3) | 0 (0) | 1.000 | -0.3 (-0.9, 0.3) |
| Renal Insufficiency (n, %) | 0 (0) | 0 (0) | -- | -- |
| Acute Renal Failure (n, %) | 0 (0) | 0 (0) | -- | -- |
| Cerebrovascular Accident (n, %) | 0 (0) | 0 (0) | -- | -- |
| Cardiac Arrest Requiring CPR (n, %) | 0 (0) | 0 (0) | -- | -- |
| Myocardial Infarction (n, %) | 0 (0) | 1 (0.3) | 1.000 | 0.3 (-0.3, 0.9) |
| Sepsis (n, %) | 0 (0) | 0 (0) | -- | -- |
| Septic Shock (n, %) | 0 (0) | 0 (0) | -- | -- |
| Unplanned Intensive Care Unit Admission (n, %) | 1 (0.3) | 2 (0.6) | 1.000 | 0.3 (-0.8, 1.4) |
| Mortality (n, %) | 0 (0) | 1 (0.3) | 1.000 | 0.3 (-0.3, 0.9) |

Abbreviations: **n:** sample size; **%:** percentage; **--:** not available; **>:** greater than; **CPR:** cardiopulmonary resuscitation; **MD:** mean difference, **RD%:** risk difference percentage, **95CI:** 95 percent confidence interval.

***Supplementary Table 4.*** Temporal Sensitivity Analysis of 30-Day Postoperative Outcomes, Including Early Balloon Removal, Following Intragastric Balloon (IGB) Placement (2020-2023 Cohort), Stratified by Diabetes Status After Propensity Score Matching.

| **Perioperative Parameters** | **No Diabetes (n = 111)** | **Diabetes (n = 111)** | **p-value** | **MD (95CI)** |
| --- | --- | --- | --- | --- |
| Procedure Length (mean) | 12.9 | 12.3 | 0.691 | -0.6 (-3.6, 2.4) |
| **30-Day Healthcare Utilization** | **No Diabetes (n = 111)** | **Diabetes (n = 111)** | **p-value** | **RD% (95CI)** |
| Outpatient Intravenous Treatment (n, %) | 8 (7.2) | 8 (7.2) | 1.000 | 0.0 (-6.8, 6.8) |
| Greater Than One Day From Procedure to Discharge (n, %) | 2 (1.8) | 3 (2.7) | 1.000 | 0.9 (-3.0, 4.8) |
| Emergency Department Visit (n, %) | 6 (5.4) | 10 (9.0) | 0.299 | 3.6 (-3.2, 10.4) |
| Readmission (n, %) | 2 (1.8) | 6 (5.4) | 0.280 | 3.6 (-1.3, 8.5) |
| Reoperation (n, %) | 2 (1.8) | 1 (0.9) | 1.000 | -0.9 (-4.0, 2.0) |
| Intervention (n, %) | 9 (8.1) | 7 (6.3) | 0.604 | -1.8 (-8.6, 5.0) |
| Balloon Removal (n, %) | 5 (4.5) | 7 (6.3) | 0.768 | 1.8 (-4.1, 7.7) |
| **30-Day Postoperative Serious Adverse Events** | **No Diabetes (n = 111)** | **Diabetes (n = 111)** | **p-value** | **RD% (95CI)** |
| Organ Space Infection (n, %) | 0 (0) | 0 (0) | -- | -- |
| Pneumonia (n, %) | 0 (0) | 0 (0) | -- | -- |
| Unplanned Intubation (n, %) | 1 (0.9) | 0 (0) | 1.000 | -0.9 (-2.7, 0.9) |
| Pulmonary Embolism (n, %) | 0 (0) | 0 (0) | -- | -- |
| Deep Vein Thrombosis (n, %) | 0 (0) | 0 (0) | -- | -- |
| On Ventilator > 48 hours (n, %) | 0 (0) | 0 (0) | -- | -- |
| Urinary Tract Infection (n, %) | 0 (0) | 0 (0) | -- | -- |
| Renal Insufficiency (n, %) | 0 (0) | 0 (0) | -- | -- |
| Acute Renal Failure (n, %) | 0 (0) | 0 (0) | -- | -- |
| Cerebrovascular Accident (n, %) | 0 (0) | 0 (0) | -- | -- |
| Cardiac Arrest Requiring CPR (n, %) | 1 (0.9) | 0 (0) | 1.000 | -0.9 (-2.7, 0.9) |
| Myocardial Infarction (n, %) | 0 (0) | 0 (0) | -- | -- |
| Sepsis (n, %) | 0 (0) | 0 (0) | -- | -- |
| Septic Shock (n, %) | 0 (0) | 0 (0) | -- | -- |
| Unplanned Intensive Care Unit Admission (n, %) | 0 (0) | 0 (0) | -- | -- |
| Mortality (n, %) | 1 (0.9) | 0 (0) | 1.000 | -0.9 (-2.7, 0.9) |

Abbreviations: **n:** sample size; **%:** percentage; **--:** not available; **>:** greater than; **CPR:** cardiopulmonary resuscitation; **MD:** mean difference, **RD%:** risk difference percentage, **95CI:** 95 percent confidence interval.

***Supplementary Table 5.*** Standard Mean Differences After Propensity Score Matching of 19 Matched Covariates for Temporal Sensitivity Analysis from 2016 to 2019 Comparing Cohorts With and Without Diabetes Undergoing Intragastric Balloon (IGB) Placement.

| **Covariate** | **Standard Mean Difference** |
| --- | --- |
| Female Sex | 0.014 |
| Age Greater than 45 | 0.021 |
| Non-Hispanic White | 0.053 |
| Body Mass Index Greater than 35 | 0.060 |
| Smoker within One Year | 0.038 |
| Immunosuppressant Use | 0.047 |
| Chronic Obstructive Pulmonary Disease | < 0.001 |
| History of Pulmonary Embolism | 0.066 |
| Sleep Apnea | 0.028 |
| Gastroesophageal Reflux Disease | 0.069 |
| Previous Surgery | 0.013 |
| History of Myocardial Infarction | 0.030 |
| Prior Percutaneous Coronary Intervention | 0.102 |
| Hypertension | 0.013 |
| Hyperlipidemia | 0.007 |
| History of Deep Vein Thrombosis | 0.046 |
| Venous Stasis | 0.113 |
| Therapeutic Anticoagulation Use | 0.115 |
| Renal Insufficiency | 0.036 |
| Overall Match Distance | |
| Pre-Match Distance | Post-Match Distance |
| 0.738 | 0.025 |

***Supplementary Table 6.*** Standard Mean Differences After Propensity Score Matching of 19 Matched Covariates for Temporal Sensitivity Analysis from 2020 to 2023 Comparing Cohorts With and Without Diabetes Undergoing Intragastric Balloon (IGB) Placement.

| **Covariate** | **Standard Mean Difference** |
| --- | --- |
| Female Sex | 0.065 |
| Age Greater than 45 | 0.072 |
| Non-Hispanic White | 0.093 |
| Body Mass Index Greater than 35 | 0.055 |
| Smoker within One Year | < 0.001 |
| Immunosuppressant Use | < 0.001 |
| Chronic Obstructive Pulmonary Disease | < 0.001 |
| History of Pulmonary Embolism | 0.078 |
| Sleep Apnea | 0.090 |
| Gastroesophageal Reflux Disease | 0.096 |
| Previous Surgery | < 0.001 |
| History of Myocardial Infarction | < 0.001 |
| Prior Percutaneous Coronary Intervention | 0.078 |
| Hypertension | 0.036 |
| Hyperlipidemia | 0.108 |
| History of Deep Vein Thrombosis | < 0.001 |
| Venous Stasis | 0.134 |
| Therapeutic Anticoagulation Use | < 0.001 |
| Renal Insufficiency | < 0.001 |
| Overall Match Distance | |
| Pre-Match Distance | Post-Match Distance |
| 0.713 | 0.028 |

***Supplementary Table 7.*** Missing Data Analysis Comparing Baseline Demographics and Preoperative Associated Medical Problems Stratified by Availability of HbA1c Data.

| **Demographics** | **HbA1c Data Unavailable (n = 4,043)** | **HbA1c Data Available (n = 512)** | **p-value** |
| --- | --- | --- | --- |
| Age (mean) | 45.8 | 46.3 | 0.364 |
| Body Mass Index (mean) | 33.6 | 35.3 | 0.002 |
| Female Sex (n, %) | 3,328 (82.3) | 423 (82.6) | 0.866 |
| Non-Hispanic White (n, %) | 2,608 (64.5) | 331 (64.6) | 0.950 |
| Non-Hispanic Black (n, %) | 389 (9.6) | 58 (11.3) | 0.221 |
| Hispanic Ethnicity (n, %) * | 328 (9.3) | 40 (8.6) | 0.599 |
| **Preoperative Associated Medical Problems** | **HbA1c Data Unavailable (n = 4,043)** | **HbA1c Data Available (n = 512)** | **p-value** |
| Smoker within One Year (n, %) | 203 (5.0) | 21 (4.1) | 0.365 |
| Chronic Obstructive Pulmonary Disease (n, %) | 7 (0.2) | 1 (0.2) | 1.000 |
| History of Pulmonary Embolism (n, %) | 17 (0.4) | 0 (0) | 0.246 |
| History of Deep Vein Thrombosis (n, %) | 12 (0.3) | 4 (0.8) | 0.096 |
| Therapeutic Anticoagulation Use (n, %) | 33 (0.8) | 4 (0.8) | 1.000 |
| Previous Surgery (n, %) | 192 (4.7) | 9 (1.8) | 0.002 |
| Sleep Apnea (n, %) | 419 (10.4) | 65 (12.7) | 0.110 |
| Gastroesophageal Reflux Disease (n, %) | 936 (23.2) | 133 (26.0) | 0.166 |
| Hypertension (n, %) | 1,015 (25.1) | 147 (28.7) | 0.078 |
| Hyperlipidemia (n, %) | 520 (12.9) | 85 (16.6) | 0.019 |
| Venous Stasis (n, %) | 10 (0.2) | 0 (0) | 0.615 |
| History of Myocardial Infarction (n, %) | 17 (0.4) | 2 (0.4) | 1.000 |
| Prior Percutaneous Coronary Intervention (n, %) | 32 (0.8) | 4 (0.8) | 1.000 |
| Previous Cardiac Surgery (n, %) | 21 (0.5) | 5 (1.0) | 0.205 |
| Inferior Vena Cava Filter (n, %) | 2 (0.0) | 0 (0) | 1.000 |
| Renal Insufficiency (n, %) | 5 (0.1) | 4 (0.8) | 0.013 |
| Dialysis (n, %) | 3 (0.1) | 0 (0) | 1.000 |
| Independent Functional Status (n, %) * | 4,025 (99.9) | 510 (99.6) | 0.225 |
| ASA Class Greater Than 2 (n, %) * | 3,610 (94.5) | 442 (95.1) | 0.745 |

Abbreviations: **HbA1c:** hemoglobin A1c, **n:** sample size; **%:** percentage; **ASA:** American Society of Anesthesiologists. *Denotes missing data within the covariate less than the overall sample size listed.

## *Supplementary Table 8.* Statistical Power, Detectable Difference Analysis for Rare Binary Outcomes Based on Matched Cohort Sizes.

| **Matched Cohort Size** | **Baseline Event Rate (%)** | **Detectable Comparator Rate (%)** | **Minimum Detectable Absolute Difference (%)** |
| --- | --- | --- | --- |
| 424 vs 424 | 0.2 | 2.36 | 2.16 |
| 424 vs 424 | 0.5 | 3.03 | 2.53 |
| 424 vs 424 | 1.0 | 4.00 | 3.00 |
| 424 vs 424 | 2.0 | 5.69 | 3.69 |
| 106 vs 106 | 0.2 | 7.62 | 7.42 |
| 106 vs 106 | 0.5 | 8.38 | 7.88 |
| 106 vs 106 | 1.0 | 9.56 | 8.56 |
| 106 vs 106 | 2.0 | 11.65 | 9.65 |

**Detectable comparator rate**: the event rate in the comparison group required to achieve 80% statistical power for detection, **minimum detectable absolute difference:** the minimum detectable between-group difference, **α** = 0.05.
